# Polarity Related Influence Maximization in Signed Social Networks

**DOI:** 10.1371/journal.pone.0102199

**Published:** 2014-07-25

**Authors:** Dong Li, Zhi-Ming Xu, Nilanjan Chakraborty, Anika Gupta, Katia Sycara, Sheng Li

**Affiliations:** 1 School of Computer Science and Technology, Harbin Institute of Technology, Harbin, Heilongjiang, China; 2 School of Computer Science, Carnegie Mellon University, Pittsburgh, Pennsylvania, United States of America; Universitat Rovira i Virgili, Spain

## Abstract

Influence maximization in social networks has been widely studied motivated by applications like spread of ideas or innovations in a network and viral marketing of products. Current studies focus almost exclusively on unsigned social networks containing only positive relationships (e.g. friend or trust) between users. Influence maximization in signed social networks containing both positive relationships and negative relationships (e.g. foe or distrust) between users is still a challenging problem that has not been studied. Thus, in this paper, we propose the polarity-related influence maximization (PRIM) problem which aims to find the seed node set with maximum positive influence or maximum negative influence in signed social networks. To address the PRIM problem, we first extend the standard Independent Cascade (IC) model to the signed social networks and propose a Polarity-related Independent Cascade (named IC-P) diffusion model. We prove that the influence function of the PRIM problem under the IC-P model is monotonic and submodular Thus, a greedy algorithm can be used to achieve an approximation ratio of 1-1/e for solving the PRIM problem in signed social networks. Experimental results on two signed social network datasets, Epinions and Slashdot, validate that our approximation algorithm for solving the PRIM problem outperforms state-of-the-art methods.

## Introduction

Online social networks such as Twitter, Facebook and Google+ have developed rapidly in recent years. They support social interaction and information diffusion among users all over the world. These online sites present great opportunities for large-scale viral marketing. Viral marketing, first introduced to the data mining community by Domingos and Richardson [1], is a cost-effective marketing strategy that promotes products by giving free or discounted items to a selected group with high influence, in the hope that through the word-of-mouth effects, a large number of users will adopt the product. Motivated by viral marketing, influence maximization emerges as a fundamental problem concerning the diffusion of products, opinions, and innovations through social networks [2].

Influence maximization has been formulated as a discrete optimization problem by Kempe et al. [3]. Given a social network modeled as a graph 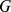, find 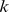 nodes, such that by activating them initially, the expected number of nodes activated by these 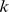 seed nodes is maximized under a certain diffusion model. Diffusion models are used to explain and simulate the spread of information in social networks. Two widely used diffusion models are the Independent Cascade (IC) model and Linear Threshold (LT) model. Based on these diffusion models and their extensions, influence maximization problem have been extensively studied [2], [4]–[9], where improved greedy algorithms and scalable heuristics are proposed to solve the problem. All the above works consider influence maximization in unsigned social networks which only have positive relationships between users (e.g. friend or trust). Actually, however, the polarity of relationships in social networks is not always positive. There are also signed social networks containing both positive relationships and negative relationships (e.g., foe or distrust) simultaneously. Influence maximization in signed social networks is a key problem that has not been studied and it is the focus of this paper.

Signed social networks can be divided into two categories: explicit networks and implicit networks. In the explicit networks, users can directly tag the polarity (positive or negative) to the relationship between two users. For example, participants on Epinions can explicitly express trust or distrust of others; users on Slashdot can declare others to be either friends or foes. In the implicit networks, users do not directly mark the polarities of relationships. However, the relationship polarities can be mined from the interaction data between users. For example, in Twitter, a user 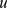 may support some of users he follows (positive) and be against the others (negative). So the relationship of "following" between users in Twitter can have polarity. The problem of turning unsigned social networks to signed social networks has been studied by several works, such as [10], [11].

**Figure 1:**
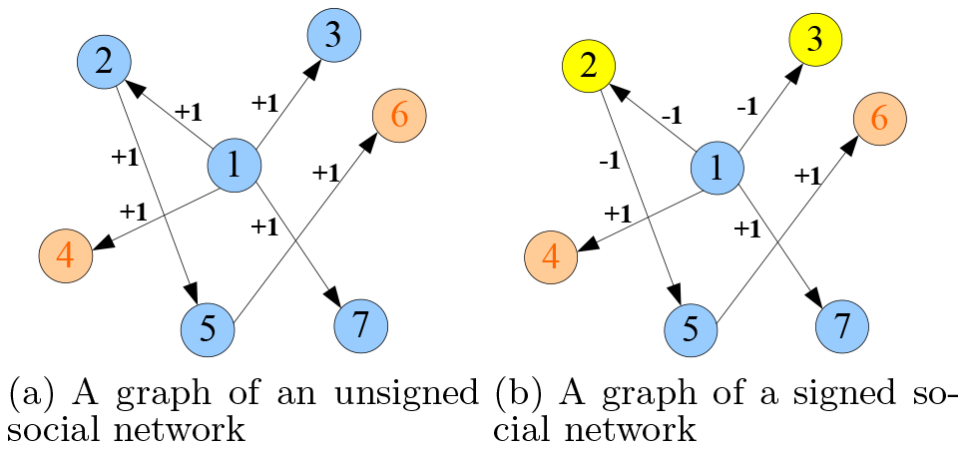
Examples of unsigned and signed social network graph.

For influence maximization in signed social networks, ignoring the relationship polarity between users to treat the signed social networks as unsigned ones and applying traditional influence maximization methods may lead to over-estimation of positive influence in practical applications. Here, we take Figure 1 and the application of viral marketing as an example to illustrate this problem of over-estimating influence. In Figure 1, three colors of nodes denote three states of users in social networks: positive, negative and inactive, which can be understood as promoting, opposing and not caring about the product in viral marketing application. Blue means positive state, yellow means negative state and brick red means inactive state. On the edges, "+1" means positive influence relation between two nodes while "-1" means negative influence relation. For a node set, we define its positive influence as the number of nodes activated to be positive by this node set, and negative influence as the number of nodes activated to be negative by this node set. In signed networks, there are both positive and negative relationships. The initially selected nodes (e.g., node 1 in Figure 1(b)) can activate other nodes to be either positive state or negative state, and thus have positive and negative influence simultaneously. In contrast, in unsigned networks, all the relations between users are positive. Therefore, the selected nodes (e.g., node 1 in Figure 1(a)) can only activate other nodes to positive state and only have positive influence. If a signed social network is roughly treated as an unsigned social network, both the positive influence and negative influence will be mistakenly counted as positive influence. As shown in Figure 1(a) the number of nodes positively influenced by selecting node 1 will be estimated to be 5 while the actual number is 3 (as shown in Figure 1(b)). In this way, in the viral marketing, if we select the users who have large negative influence (mistaken as large positive influence) to promote the product, as a result, a lot of users will be influenced to dislike and oppose the product.

To fill the gap in the research of influence maximization in signed social networks, we propose the polarity related influence maximization (PRIM) problem. The purpose of the PRIM problem is to find the node set with maximum positive influence or maximum negative influence in signed social networks. Traditional influence maximization studies are mainly based on several classical diffusion models, such as IC model and LT model, which are only applicable to unsigned social networks but not adequate for signed social networks. Therefore, in this work, we extend the classic IC model to signed social networks. In this paper, we make the following contributions:

- We propose a novel Polarity-related Independent Cascade (IC-P) diffusion model for signed social networks. The new IC-P model incorporates the social principles that "the friend of my enemy is my enemy" and "the enemy of my enemy is my friend".
- We propose the polarity related influence maximization (PRIM) problem for the signed social networks. The PRIM is divided into two sub-problems: positive influence maximization (PIM) problem and negative influence maximization (NIM) problem.
- We prove that the influence functions of the PIM problem and NIM problem under the IC-P model of information diffusion are monotone and submodular, which allows a greedy algorithm to achieve an approximation ratio of 1-1/e.
- We conduct experiments on Epinions and Slashdot datasets. The comparison results with closely related work indicate the superiority of our method.

This paper is organized as follows: In Section 2 we discuss the related work. In Section 3 we introduce the proposed IC-P diffusion model, define the PRIM problem, prove that the influence functions of the PIM and NIM problems under IC-P diffusion model are monotone and submodular, and presents the greedy algorithm. In Section 4 we present experimental results that validate the effectiveness of our method. Finally, in Section 5 we present our conclusions and outline avenues of future research.

## Related work

In this section, we review the related work from three aspects: influence maximization problem, signed social networks, and competitive influence maximization.

### Influence maximization (IM) problem

Domingos and Richardson were the first to consider the IM problem as an algorithmic problem [1], [12], where they model the social networks as markov random fields. Kempe et al. first formulated the problem as a discrete optimization problem in [3]. The authors proved that the optimization problem of selecting the most influential nodes is NP-hard, and presented a greedy approximation algorithm which is applicable to the IC model and LT model. However, the greedy algorithm in [3] is not scalable.

Several recent studies aimed at addressing this scalability issue. Kimura and Saito proposed shortest-path based influence cascade models and provided efficient algorithms under these models [13]. In [4], Leskovec et al. presented an optimization in selecting new seeds, which was referred to as the "Cost-Effective Lazy Forward" (CELF) scheme. The CELF optimization used the submodularity property. Chen et al. proposed a scalable heuristic called LDAG for the LT model [6]. They constructed local directed acyclic graphs (DAGs) for each node and considered influence only within it. More recently, Chen et al. proposed Prefix excluding Maximum Influence Arborescence (PMIA) heuristic to estimate influence spread under the IC model [5].

Goyal et al. proposed an alternative approach which, instead of assuming influence probabilities are given as input, directly uses the past available data [7]. Liu et al. and Chen et al. studied the time constrained influence maximization problem [2], [14]. Narayanam and Narahari proposed a new way of solving these problems using the concept of Shapley value which is a well known solution concept in cooperative game theory [15]. However, the above works do not consider the influence maximization for signed social networks.

### Signed social networks

The signed social networks containing both positive relationships and negative relationships have attracted increasing attention. Brzozowski et al. studied the positive and negative relationships that exist on ideologically oriented sites such as Essembly, with the goal of predicting outcomes of group votes rather than the broader organization of the social network [16]. Kunegis et al. studied the friend/foe relationships on Slashdot, and computed global network properties [17]. They also studied signed spectral clustering methods, signed graph kernels and network visualization methods in signed graphs [18]. Leskovec et al. connected their analysis to theories of signed networks from social psychology [19]. Another study of Leskovec et al. used signed triads as features and constructed a logistic regression model for predicting positive and negative links [20]. Ye et al. adopted the transfer learning approach to leverage the edge sign information from the source network for predicting the positive and negative links [21]. Yang et al. studied the problem of turning an unsigned acquaintance network (e.g. Facebook, Myspace) into a signed trust-distrust network [10]. Facchetti et al. analyzed the structural balance in large signed networks. They concluded that most on-line networks available today exhibit structural balance [22]. Fan et al. extended the Susceptible Infected Recovered (SIR) model from epidemiology to signed networks, to model the process of opinion diffusion in signed networks [23]. However, none of above works deal with the problem of influence maximization in signed social networks.

### Competitive influence maximization

Here, the extant literature researches [24]–[28] usually extend the classical diffusion models, such as IC model and LT model, to the situation where two or more competitive messages spread in the social network simultaneously. They study how to select a fixed number of nodes that maximize influence for different competitive messages. However, all these works are limited to unsigned social networks. In contrast, we extend the IC model to signed social networks, and study influence maximization problem in signed social networks based on the proposed diffusion model.

## Materials and Methods

In this section, we first introduce how to model a signed social network as a directed and signed graph, and then propose the diffusion model on the directed and signed graphs. Next, we define the polarity related influence maximization (PRIM) problem, and prove properties of the influence function in PRIM problem. At last, we propose greedy algorithm to solve PRIM problem.

### Modeling Signed Social Networks

An unsigned social network can be modeled as a directed graph 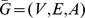, where 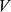 is the set of nodes, and 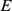 is the set of directed edges. Nodes and edges in the graph correspond to users and relationships between users in the social networks, respectively. 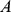 is a non-negative weighted adjacency matrix with 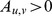 if and only if the edge 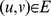, with 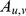 as its weight. Different from unsigned social networks, in this paper, we model a signed social network as a directed and signed graph 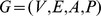, where 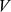, 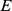, 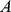 are defined exactly as in the graph 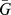. Additionally, 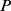 is a matrix whose element 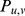 is the sign of edge 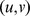 in the graph. Note that in the directed and signed graph 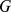, the relations between nodes are asymmetric, i.e. 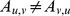 and 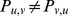.

**Figure 2:**
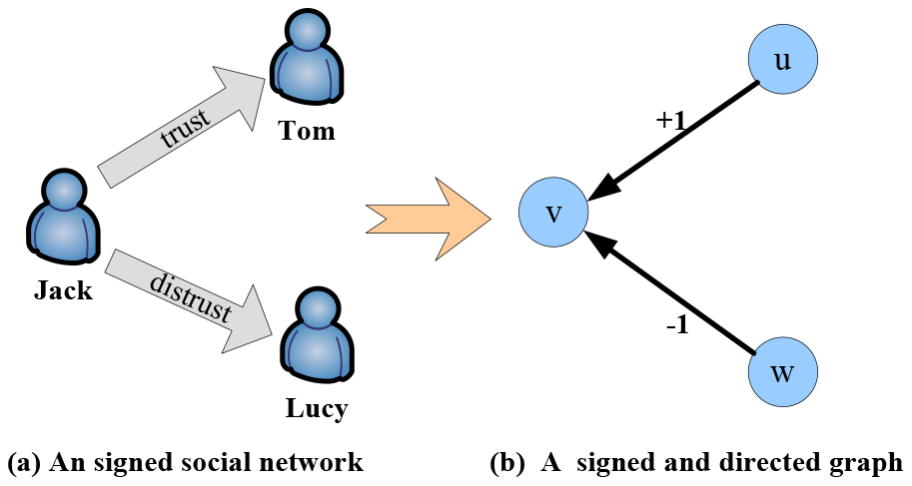
An example of modeling a signed social network.

Here we take Figure 2 as an example to explain the modeling process of signed social networks. Figure 2(a) shows an example of a signed social network which contains three users (Jack, Tom and Lucy) and two relationships among them. Figure 2(b) presents the graph model of the signed social network in Figure 2(a). Three nodes 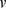, 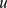 and 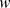 are corresponding to user Jack, Tom and Lucy respectively. The edges in the graph correspond to social relationships among the three users. Here, we should note that the direction of an edge in the graph is the opposite of social relationship in the social network. For example, the social relationship is from Jack to Tom in Figure 2(a), while the corresponding edge is from 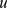 to 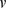 in Figure 2(b). This is because the graph we modeled is a influence diffusion graph, and the direction of influence spreading between users is opposite to that of the social relationship between them. If there is a relationship from Jack to Tom, influence spreads from Tom to Jack, which means Jack is influenced by Tom, so the edge should be 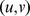 but not 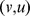 in the graph. Finally, the signs of edges in the graphs correspond to polarities of social relationships between users. In Figure 2, Jack trusts Tom, so 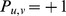; Jack distrusts Lucy, so 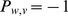; there is no relationship between Tom and Lucy, so 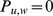. The values on the edges in Figure 2(b) are the signs but not weights of edges. In the context of influence diffusion, the weight 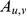 can be considered as the influence diffusion probability from 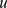 to 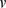, which can be calculated based on interactive data between users or assigned by some weight models [3], [5], [6].

### Polarity-related Diffusion Model

We first describe the standard Independent Cascade (IC) model for information diffusion in [3] used in unsigned social networks. In the IC model, each node in the graph has two states, active or inactive. For a node 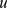, the active state can be considered as the state where the corresponding user in the social network adopts the information (e.g., product or idea) spreading in the network. Inactive state of 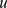 can be considered as the state where the corresponding user does not adopt the information. The diffusion process starts with an initial set of active nodes 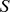, and unfolds in discrete steps according to the following randomized rule. In the step 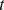, any node 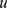 activated at step 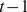 is given a single chance to activate each of its currently inactive neighbors 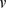; it succeeds with a probability 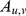 independently. If 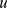 succeeds, then 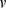 will become active in step 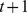. But whether or not 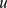 succeeds, it can not make any further attempts to activate 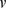 in subsequent rounds. The process runs until no more activations are possible. If node 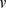 has multiple newly activated neighbors in a time step, their activation attempts are sequenced in an arbitrary order.

In this paper, based on the social principles that "the friend of my enemy is my enemy" and "the enemy of my enemy is my friend", we propose Polarity-related Independent Cascade (named IC-P) diffusion model which incorporates the polarity of relationship between users in signed social networks. The IC-P model is an extension of the IC model to signed social networks. In the IC-P model, the active state of nodes is divided into positive state and negative state. Therefore, each node in the IC-P model has three states: positive, negative, or inactive. For a node 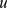, positive state means that, in the social network, the corresponding user adopts and then supports or trusts the spreading information. Negative state of 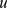 means that the corresponding user adopts but then opposes or distrusts the information. Inactive state of 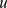 means that the corresponding user does not adopt the information. We use 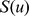 to denote the state of node 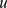, and values 1, −1, 0 of 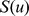 to denote 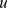
's positive state, negative state and inactive state, respectively.

In the IC-P model, the diffusion process starts with an initial set of active nodes 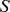. 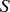 can contain both positive nodes and negative nodes. All other nodes not in 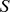 are inactive in the graph. The process unfolds in discrete steps according to the following randomized rule. For a node 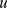 activated in time step 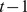, it will become positive or negative state in time step 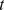. Then this node 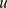 will have a single chance to activate each currently inactive neighbor 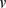 in time step 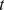. For a node 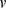, we define 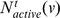 as the neighbor set of node 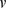 who become positive or negative in time t. In time 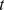, each node 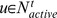 will activate 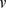 successfully with the probability 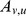 in an arbitrary order. Once the node 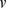 is activated by a node in 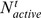, other nodes in 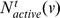 can not activate node 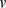 any more. In the proposed IC-P model, a node 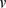 can only be activated once in a time step, which is different from standard IC model.

For a newly activated node 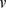, its state 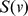 is related to the state of the node 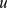 that activated 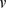 and the polarity of relation between node 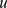 and 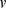, that is, 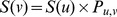. Therefore, if node 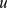 is positive and the relation between 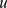 and 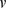 is positive, then the node 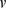 will become positive. If node 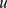 is negative and the relation between 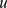 and 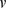 is positive, then the node 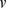 will become negative. If node 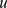 is positive and the relation between 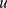 and 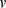 is negative, then the node 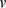 will become negative. If node 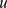 is negative and the relation between 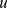 and 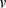 is negative, then the node 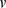 will become positive. Once a node becomes positive or negative, it will not change its state any more. The process continues until there is no newly activated node.

In the IC-P model, once a node becomes positive or negative, it will not change its state in the future. So our model is not a susceptible-infected-susceptible (SIS) type diffusion model, in which the susceptible node could be infected and become susceptible again later. Here, we explain why we do not design our model as SIS type. In a SIS type diffusion model designed for signed social networks, there would be a situation like this: 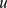 is an initial seed node, it is positive (support an opinion or a product) at the beginning, then 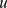 will try to activate its neighbors to be positive or negative. After some time, 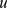 becomes susceptible but some of his neighbors are still infected, then its neighbor may attempt to activate 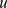 to be negative conversely. So 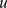 may be activated to be negative in the end. That is, 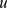 supports the opinion or product at the initial time but opposes it in the end. This does not meet practical scenarios. For example, in the application of viral marketing, the company pays some initial users to let them support its product. If we adopt a SIS type model, some paid initial users may become to oppose the product in the end of diffusion process, which is illogical. The SIS type models are more suitable to simulate epidemic diffusion than information diffusion. Because, epidemic diffusion is undirected: epidemic can spread between two linked users for many times. Differently, information diffusion is directed: an active user tries to activate inactive user, and the activated user should not attempt to activate the user who activates him. Current diffusion models used for solving influence maximization problem are mainly independent cascade model, linear threshold model and their various extensions [2], [3], [6], [26], [28]. All these models are not SIS type. Our model can be considered as an extension of traditional Independent Cascade model. SIS type diffusion models are rarely used in research of influence maximization problem.

To demonstrate the rationality of our model, we discuss in more details the applicability of the proposed model to real processes. Here we take the opinions promotion and viral marketing as examples for discussions. In the opinions promotion application, political candidates try to find supporters for their political opinions. Given a signed social network about political relations, positive relations represent political allies and negative relations represent political enemies. In our proposed model, when a person supports a political opinion, his political allies will also support the political opinion, and his political enemies will opposite the political opinion, and the political enemies of his political enemies will support the political opinion. In the viral marketing application, companies try to find early adopters to promote their products. Given a signed social network about production, positive relations represent trust people and negative relations represent distrust people. In our proposed model, when a person adopts a product, people who trust him will also adopt the product, and people who distrust him will not adopt the product, and people distrusting the people who distrust him will adopt the product.

Our model is not perfect currently, it only focuses on the impact of polarity social relation on information diffusion, and does not consider the polarity of information content. So, our model is content-independent. Applications such as political struggle and product adoption are also actually more complicated than the simulation process of our model. In political struggle, a politician may support his enemy's opinion for political benefits. In product adoption, a user may not care what product is adopted by his distrusted user. Our work is the first attempt to model information diffusion over signed social networks, and we will improve it for more accurate simulation of the real world.

### PRIM Problem Definition

Influence maximization is the problem of finding a small subset of seed nodes in a network graph, given a diffusion model, that could maximize the spread of influence. Current studies [3]–[6], [13], only focus on unsigned social networks which only have positive relationships. However, Influence maximization in signed social networks containing both positive relationships and negative relationships is still a challenging problem that has not received much attention. Therefore, based on the IC-P diffusion model we proposed, we propose the polarity-related influence maximization (PRIM) problem which takes the polarity of relations in signed social networks into account, and can achieve more competent result in viral marketing.

Let 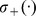 be the positive influence function. Given an initial node set 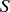, 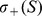 returns the positive influence of 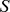, and the returned value is the expected number of nodes activated to be positive by 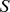 based on the IC-P diffusion model. Similarly, 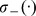 is defined as the negative influence function and 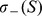 returns the negative influence of 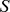, and the returned value is the expected number of negative nodes activated by 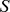 based on the IC-P model. Besides, we also define 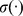 as the non-polar influence function, and 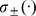 as the net positive influence, i.e., for the node set 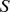, 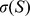  =  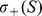 + 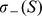, 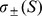  =  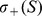 − 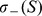.

Given the graph of a signed social network 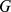 and a non-negative number 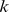, based on the IC-P diffusion model, the PRIM problem is to find a set 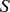 of 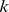 seed nodes such that the expected number of positive nodes 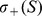 is maximized or the expected number of negative nodes 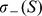 is maximized. Without loss of generality, all seed nodes in the initial set 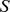 are assumed to be positive. Therefore, based on above definition, the PRIM problem can be divided into two sub-problems, positive influence maximization (PIM) problem and negative influence maximization (NIM) problem.

PIM problem is to find the node set with maximum positive influence, which can be formalized as,
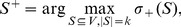
(1)


NIM problem is to find the node set with maximum negative influence, which can be formalized as,
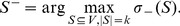
(2)


The studies of the PIM and NIM problems have extensive application scenarios. PIM can be applied to viral marketing, and companies or individuals can use it to promote their products, services and innovative ideas. NIM can be combined with the study of PIM for the situation where more than one competitive information spread in the social networks simultaneously. For two competitive information 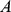 and 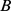, if we want to support 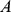 but oppose 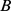, we can choose the node set selected by PIM to promote 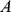, and choose the node set selected by NIM to promote 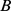.

Without loss of generality, all seed nodes in the initial set 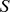 are assumed to be positive in the PIM and NIM problem. This assumption is designed based on the particular application scenarios of our proposed problem. We take the PIM problem and its application of viral marketing as an example. PIM problem applied in viral marketing is to find the node set with maximum positive influence to promote one product in a signed social network. In this application scenario, the initial seed node set has two options. The first one is only containing positive nodes, and the other one is containing both positive and negative nodes. The later option means that the company chooses some people and pays them to release negative opinion about its product for promoting. This is unreasonable. Therefore, the second option is not applicable to this application scenario. We will explore appropriate application scenarios for the second option in future work, and illustrate our proposed IC-P model in those contexts. In the PIM problem we defined, though all initial seed nodes are positive, there are negative relations in signed social networks and they will lead negative opinions happen.

### Properties of the Influence Function

We first prove that influence function 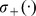 in PIM problem and influence function 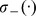 in NIM problem has the properties of monotonicity and submodularity. Then, based on the research of Nemhauser et al. [29], [30], we adopt the greedy hill-climbing algorithm to solve the PIM and NIM problems. For monotone and submodular functions, the greedy hill-climbing algorithm of starting with the empty set, and repeatedly adding an element that gives the maximum marginal gain approximates the optimum solution within a factor of 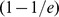. The proofs for two influence functions are similar, so we state the details for PIM.

**Theorem 1** *In the PIM problem, the positive influence function* 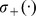 *is monotone and submodular for an arbitrary instance of the IC-P model.*

For influence function 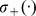 and node set 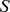, 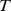, if 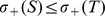 whenever 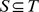, then 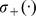 is monotone. 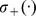 is said to the submodular if it satisfies a natural "diminishing returns" property: 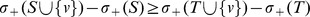, for all nodes 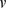 and all pairs of sets 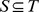, i.e., the marginal gain from adding a node to a set 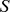 is at least as high as the marginal gain from adding the same node to a superset of 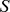.

In order to prove Theorem 1, for arbitrary sets 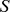 and node 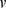, we have to firstly get the increase in value of function 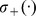 when we add 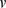 to the set 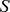, i.e., the increase of expected number of positive nodes. However, the influence diffusion in the graph under the IC-P model is a stochastic process, and the increase of positive influence is difficult to analyze directly. Kempe et al. [3] constructed the live-edge process, which is equivalent to diffusion process, for proving the monotonicity and submodularity of influence function. Here, we follow a similar approach to prove Theorem 1.

The live-edge process constructed by Kempe et al. [3] is as follows: they view an event of a newly activated node 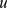 attempting to activate its neighbor 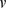 and succeeding with probability 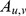 as flipping a coin with bias 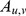. From the point of view of the process, it clearly does not matter whether the coin is flipped at the moment when 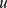 tries to activate 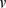, or if it was flipped at the beginning of the whole process. The edges where the coin flip indicated an activation will be successful are declared to be live; the remaining edges are declared to be blocked. Once the outcomes of the coin flips are fixed, a node 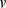 is active in diffusion process if and only if there is a path from some nodes in initial node set consisting entirely of live edges.

Different from live-edge process for IC model, in our live-edge process, the edges where coin flip is successful are only candidate-live but not live. This is because that, in the diffusion process under standard Independent Cascade (IC) model, a node can be activated for more than one times in a time step. Correspondingly, in the live-edge process, a node can have more than one live edges, and all edges where the coin flip is successful can be as viewed live. However, in the diffusion process under our proposed IC-P model, a node can only be activated for at most one time in a time step and in the whole diffusion process, the edges which are live in the live-edge process for IC model are only candidate-live (means if the start node of this directed edge were to be activated, it may succeed in activating its neighbor) in the live-edge process for IC-P model. For a node, if it has more than one candidate-live edges, we uniformly at random select one of them as the live edge, the other candidate-live edges are blocked.

Once we fix the outcomes of the coin flips, select live edge for each node and initially set all nodes in the seed set 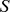 to be positive, it is clear how to determine the full set of positive nodes at the end of the cascade process:

**Claim 1** *A node* 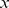 *ends up positive if and only if there is a path from one node in S to* 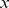 *consisting entirely of live edges, and the polarity of the path is positive. We define that* 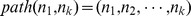 *is the live-edge path from* 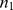 *to* 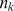 *, and the polarity of the* 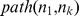 *is* 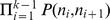 *.*

We prove that, for a node 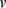, the probability of 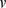 activated to be positive in diffusion process is the same as the probability of 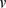 determined to be positive by the live-edge process. We define 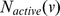  =  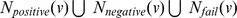 as all the active neighbors of node 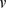 which will try to activate 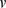, 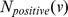 as 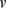
's neighbors which will activate 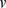 to be positive, 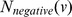 as 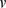
's neighbors which will activate 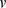 to be negative, 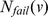 as 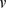
's neighbors which will fail to activate 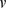. 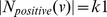, 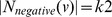, 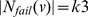.

In the diffusion process under the IC-P model, the nodes in 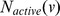 try to activate 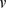 in random order, so there are totally 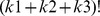 activation order choices for all nodes in 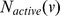. We define 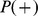 as the front-most position of all nodes belonging to 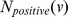 in the activation order, 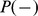 as the front-most position of all nodes belonging to 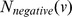 in the activation order. If 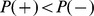 in the activation order, the node 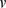 will be activated to be positive. There are 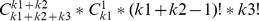 activation order choices satisfying 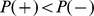, so the probability of node 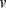 being activated to positive state is
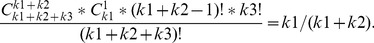
(3)


On the other hand, in the live-edge process, for node 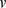, there are 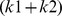 candidate-live edges. If we randomly select one from the 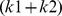 edges as live edge, the probability that the start node of the live edge belongs to 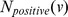 is 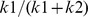. So, the probability of 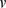 reached via positive live path is 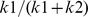, the probability of 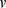 becoming positive is 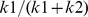 which is equal to the probability (Equ. (3)). Thus we can conclude that the live-edge process is equivalent to the diffusion process under the IC-P model.

**Proof of Theorem 1** In live-edge process for the IC-P model, after coin flipping events and live edge selecting events, each edge will have a outcome (live or blocked). Consider the probability space in which each sample point specifies one possible set of outcomes for all the edges, let 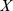 denote the set of outcomes of edges. Because we have fixed a choice for 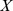, 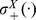 is in fact a deterministic quantity, and there is a natural way to express its value, as follows. Let 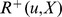 denote the set of all nodes that can be reached from 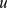 on a path consisting entirely of live edges, and the polarity of the path is positive. By Claim 1, 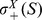 is the number of nodes that can be reached on live-edge paths from any node in 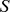, and so it equals to the cardinality of the union 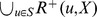.

Firstly, we prove the influence function is monotone. Obviously, 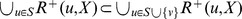, we can get 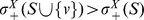, so 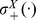 is monotone.

To see the submodularity, let 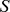 and 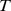 be two sets of nodes such that 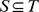. 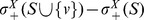 is the number of elements in 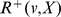 that are not already in the union 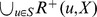, it is at least as large as the number of elements in 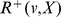 that are not in the bigger union 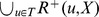, we can get
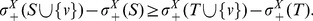
(4)

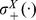
 satisfy the condition of submodular. The number of positive nodes is the weighted average over all outcomes.



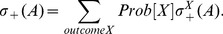
(5)A non-negative linear combination of submodular functions is also submodular, and hence 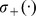 is submodular.

**Theorem 2** *In the NIM problem, the negative influence function* 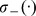 *is monotone and submodular for an arbitrary instance of the IC-P model.*

Proof of Theorem 2 is similar with that of Theorem 1. Here, we only present the Claim 2 connecting diffusion process with live-edge process for proof, omit other details.

**Claim 2** *A node* 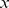 *ends up negative if and only if there is a path from one node in S to* 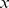 *consisting entirely of live edges, and the polarity of the path is negative.*

### Greedy Solution for PRIM

We have proved that the influence functions 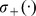 and 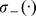 are monotone and submodular. Therefore, in this section, we use the greedy hill-climbing algorithm [29] to solve the PIM and NIM problem. Algorithm 1 presents the details of the greedy algorithm for solving the PIM problem, 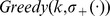, which approximates to the optimum within a factor of (1-1/e). In the algorithm 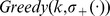, we select one node each time which provides the largest marginal increase in the function value. For the NIM problem, the greedy algorithm 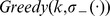 is similar with 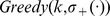.

**Table:** 

Algorithm 1 **Algorithm** 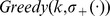 .
1: Initialize 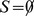 2: For 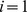 to 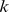 do3: select 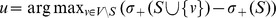 4: 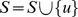 5: End for6: Output 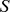 7: End

In [29], Nemhauser assumed that the greedy algorithm can evaluate the underlying function exactly. However, the number of 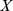 is very large in Equ(5), so it is very hard to calculate the influence value of 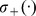 and 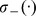 given a seed set. To mitigate this, we employ Monte Carlo simulation for estimating 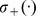 and 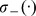 with high probability. In this case, the approximation ratio of Greedy algorithm drops to 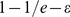, where 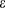 is small if the number of simulations is sufficiently large. In our experiments, we simulate 20000 times for each candidate seed node set.

Since the simulations are expensive, we adopt the CELF algorithm of Leskovec et al. [4] to reduce running time. CELF optimization utilizes submodularity such that in each round the incremental influence spread of a large number of nodes do not need to be re-evaluated because their values in the previous round are already less than that of some other nodes evaluated in the current round [31]. CELF optimization has the same influence spread as the original greedy algorithm but is much faster.

## Experiments

In this section, we conduct experiments on two real-world explicit signed social networks. The proposed algorithm is evaluated and compared with a number of state-of-the-art algorithms adopted in signed networks. The results show that the proposed algorithm under the proposed IC-P model can find the seed node set with maximum positive or negative influence more accurately than the greedy algorithm under standard IC model and other heuristic algorithms.

### Experiment Setup

#### Datasets

We use two large online signed social networks 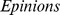 and 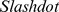, where each relationship between users is explicitly labeled as positive or negative. Both of these two networks are downloaded from Standard Large Network Dataset Collection (http://snap.stanford.edu/data/index.html). We model the two signed social networks as two signed graphs. Since the original graphs are too large, similar as the previous well-known work [21], we select two subgraphs of original data. We will evaluate the effectiveness of our method on original graphs, and do dense experiments on subgraph datasets.

- **Epinions.** This is a product review site where users choose whether to trust or distrust one another based on their ratings and reviews of products. This original network has 131,828 users and 841,372 relationships, and the subgraph network has 11567 users and 93204 relationships.
- **Slashdot.** This is a technology news site where users can rate each other as friend or foe. We treat those as positive and negative relations. This original network has 77350 users and 516575 relationships, and the subgraph network has 10966 users and 44356 relationships.

Table 1 shows the statistics on the two signed network graphs. By comparing the statistics of original graphs with those of subgraphs, we can see that they do not have much difference. In particular, the clustering coeffcients of the original graphs are nearly equal to those of subgraphs in both two datasets. We can also see that Epinions graph has a larger number of nodes, edges, average out degree, average positive out degree and clustering coefficient, while Slashdot graph has a larger number of average negative out degree. The proportion of negative relationships in Slahsdot are much higher than that in Epinions. Note that, although we use two explicit signed networks as the experiment datasets, our algorithm is also applicable to implicit signed networks where polarity of the relationship can be mined from interactive data between users.

**Table 1:** Statistics on two signed network graphs.

Dataset	Epinions (original)	Epinions (subgraph)	Slashdot (original)	Slashdot (subgraph)
Nodes	131828	11567	77350	10966
Edges	841372	93204	516575	44356
Average Out-Degree	6.38	8.06	6.68	4.04
Maximal Out-Degree	2070	429	2532	243
Average Positive Out-Degree	5.44	7.23	5.12	2.99
Maximal Positive Out-Degree	2070	428	2502	225
Average Negative Out-Degree	0.94	0.83	1.56	1.05
Maximal Negative Out-Degree	1562	182	495	123
Clustering Coefficient	0.1279	0.1269	0.0549	0.0583

#### Generating influence probabilities

Because we can not get the data to compute the influence diffusion probability (edge weight) 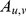 for each edge 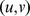 in graph 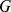, here, we adopt three models, proposed in [3], [5], [6], to generate these diffusion probabilities.

- **Weighted Cascade (WC) model.** In this model [3], 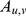 for an edge 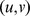 is 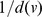, where 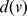 is the in-degree of 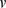.
- **TRIVALENCY model.** On each edge 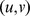, this model randomly selects a diffusion probability from the values 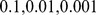, which correspond to high, medium, and low influence diffusion probabiltiy, respectively.
- **uniformly (UN) model.** All edges are uniformly assigned same probability. We will test five diffusion probabilities: 0.01, 0.02,0.03, 0.05 and 0.08.

#### Comparison methods

We compare our method called IC-P greedy with IC greedy algorithm under standard IC model and several heuristic algorithms. Following lists the algorithms we evaluate and compare in our experiments.

- **IC-P Greedy.** This is our presented method.
- **IC Greedy.** We use the original greedy algorithm under the standard IC model with the lazy-forward optimization [4] in the network graph where edges polarities are neglected, to get the seed node set of size 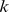 with maximum influence (non-polar).
- **Out-Degree.** This is a heuristic algorithm that selects 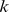 nodes with the largest out degrees, which is also evaluated in [3], [5].
- **Positive Out-Degree.** This is a heuristic algorithm that selects 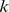 nodes with the largest positive out-degree. This algorithm is used as a baseline for the PIM problem in our experiments.
- **Negative Out-Degree.** This is a heuristic algorithm that selects 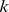 nodes with the largest negative out-degree. This algorithm is used as a baseline for the NIM problem in our experiments.
- **Random.** This method randomly selects the 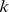 random nodes from the graph, which is also evaluated in [3], [5].

To obtain the positive or negative influence of these seed node sets selected by IC Greedy and heuristic algorithms, for each seed node set, we run the simulation using our IC-P diffusion model in the signed graphs for 20000 times, then take the average all these simulations. On the original graphs, the selected number k is set to be 20. We compare their positive influence with different sizes of seed node set, ranging from 1 to 20. On the subgraphs, the selected number k is set to be 50. We compare their positive influence or negative influence with different sizes of seed node set, ranging from 1 to 50. All the experiments are implemented on a server with 2.40GHz Six-Core Intel Xeon E5645 and 24G memory.

### Experiment Results

In this section, we summarize our experiment results involving different algorithms with different diffusion probabilities on two real life datasets. In this paper, the dataset we mention means subgraph dataset. When we use original datasets, we will particularly emphasize what we use are original graph datasets.

#### Results of the PIM problem

**Figure 3:**
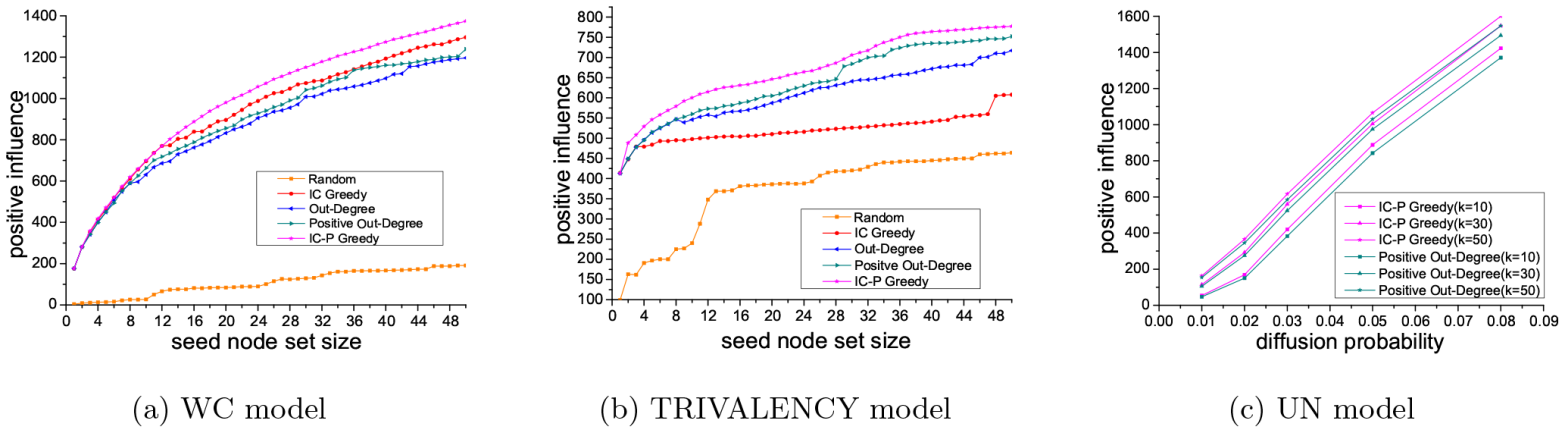
Results on Epinions dataset for PIM problem.

Figure 3 shows the performance concering the PIM problem, using five different algorithms (IC Greedy, Out-Degree, Positive Out-Degree, Random and IC-P Greedy) with three kinds of diffusion probability (WC model, UN model, TRIVALENCY model) on the Epinions dataset.

Figure 3(a) and Figure 3(b) present the positive influence of the seed node sets selected by five methods with WC model and TRIVALENCY model. The size of seed node set ranges form 1 to 50. For UN model, we compare the performance of above five algorithms with five different probabilities(0.01, 0.02, 0.03, 0.05 and 0.08). The results show that, excluding the proposed method, the positive out degree method get the best performance. Therefore, in Figure 3(c), we present the performance of our proposed IC-P Greedy and the Positive Out-Degree with UN model and the size of the seed node set is set to be 10, 30 and 50, respectively. As we can see in Figure 3, our proposed method has the best performance while the random baseline is the worst, indicating that a careful seed selection is indeed important for effective viral marketing results. Compared to IC Greedy, Positive Out-Degree and Out-Degree methods, our method is 6.0%, 10.9% and 14.9% better with WC model, and is 27.8%, 3.4% and 8.4% better with TRIVALENCY model. With UN model, our method outperforms Positive Out Degree by 3.4% when the influence probability is set to be 0.08.

**Figure 4:**
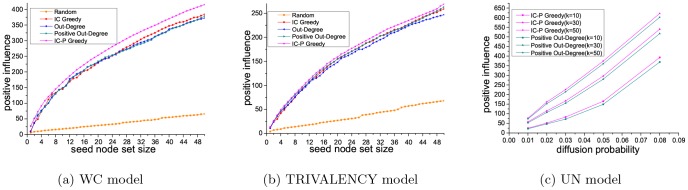
Results on Slashdot dataset for PIM problem.

Figure 4 presents the experiment results on Slashdot dataset. Similarly, the proposed method performs best and the random baseline performs worst. Compared to IC Greedy, Positive Out-Degree and Out-Degree, our method is 8.1%, 9.8% and 11.6% better with WC model, and is 6.2%, 4.7% and 11.3% better with TRIVALENCY model. With UN model, our method is 3.3% better than Positive Out-Degree when the influence probability is set to be 0.08.

From Figure 3 and Figure 4, we can see that our proposed IC Greedy algorithm performs better than Positive Out-Degree with WC model, but performs worse than Positive Out-Degree with TRIVALENCY model and UN model. In contrast, our method can constantly achieve the best performance among all the compared methods on both datasets with three kinds of diffusion probability, which indicates that our method is more stable than the others. In WC model, the diffusion probabilities are calculated based on the in-degree of nodes in graphs while in TRIVALENCY model and UN model the diffusion probabilities are randomly assigned. Therefore, obviously, the WC model is more reasonable and accurate than the other two models. By comparing the performances of our method in conjunction with these three kinds of diffusion probability, we can see that our method performs best on WC model. This result illustrates that if our method is applied to the graph fed with more accurate diffusion probability, it can achieve better performance for the PIM problem, which also confirms the rationality of the proposed method.

**Figure 5:**
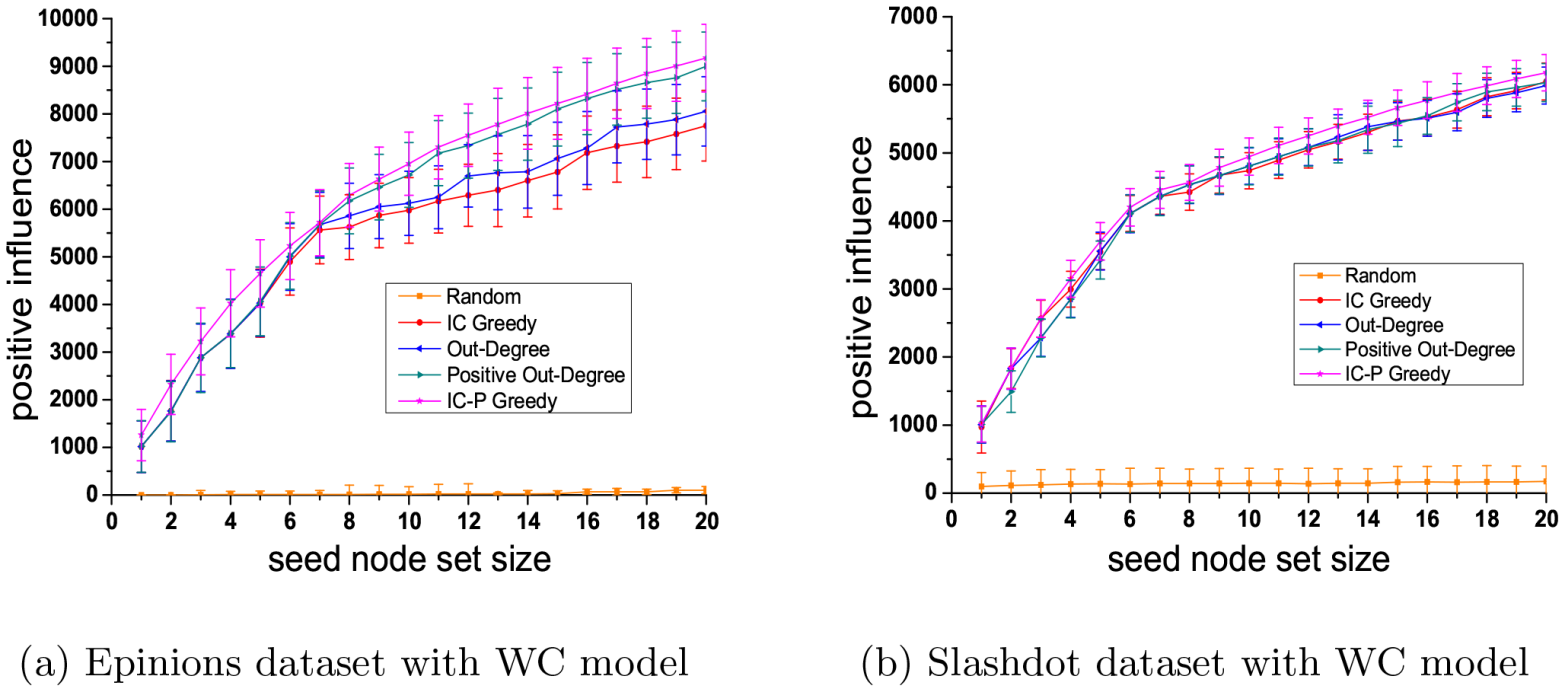
Results on original datasets for PIM problem.

For the PIM problem, we also do experiments on original graphs of Epinions dataset and Slashdot dataset. Figure 5 shows the performance concerning the PIM problem, using five different algorithms with WC model as diffusion probability on the original graphs of both the two datasets. To quantify the extent of fluctuations around the average, we also compute standard deviations and draw standard deviation bar for each influence plot. We can see that the results on original graphs are similar with those on subgraphs. Our method performs best among five methods. Therefore, we can consider that the experimental results on subgraph datasets can support the conclusion of our paper reasonably.

#### Results of the NIM problem

**Figure 6:**
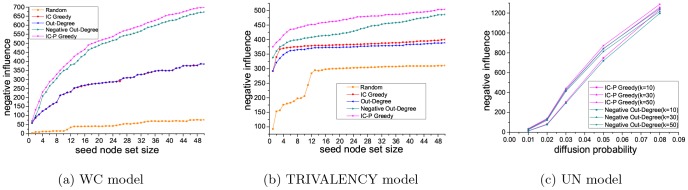
Results on Epinions dataset for NIM problem.

**Figure 7:**
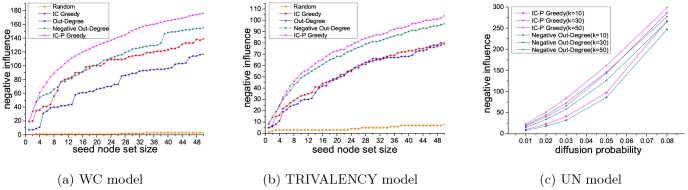
Results on Slashdot dataset for NIM problem.

Figure 6 and 7 show the performance concerning the NIM problem, using five different methods (IC Greedy, Out-Degree, Negative Out-Degree, Random and IC-P Greedy) with three kinds of diffusion probability (WC model, UN model, TRIVALENCY model) on the Epinions and slashdot datasets. As can be seen, our method achieves the best performance and random baseline method obtains the worst on both the datasets. On the Epinions dataset, comparing to IC Greedy, Negative Out-Degree and Out-Degree, our method is 81.6%, 3.9% and 81.5% better with WC model, and is 26.0%, 3.7% and 29.6% better with TRIVALENCY model. With UN model, our method is 2.7% better than Negative Out-Degree when the diffusion probability is set to be 0.08. On the Slashdot dataset, compared to IC Greedy, Negative Out-Degree and Out-Degree, our method is 26.7%, 13.5% and 50.4% better with WC model, and is 31.6%, 7.2% and 30.0% better with TRIVALENCY model. On UN model, our method is 7.6% better than Negative Out-Degree when the diffusion probability is set to be 0.08.

Above results show that, similarly with in the PIM problem, our method also achieves the best performance in solving the NIM problem. Therefore, in a word, our method can give better solution for both the PIM problem and the NIM problem compared with the baseline methods. Besides, from the results in either the PIM problem or the NIM problem, we can see the seed node set in Epinions dataset has larger influence than that in Slashdot dataset. This phenomenon is caused by the higher average out degree and clustering coefficient of the Epinions dataset.

#### Results Analysis

The seed node set has both positive influence and negative influence. In the PIM problem, we try to find a seed node set with maximum positive influence, but do not consider its negative influence. In the NIM problem, similarly, we do not consider the positive influence of the seed node set. Here, taking the PIM problem as an example, we explore the relations between positive influence and negative influence of the seed node sets selected by different methods.

PIM problem is to find the seed node set with maximum positive influence. Different methods pick out different seed node sets for solving the PIM problem. Figure 3 and Figure 4 presents the positive influence of the seed node sets selected using five different methods (IC Greedy, Out-Degree, Positive Out-Degree, Random and IC-P Greedy). Here, we also do experiments for getting non-polar influence, negative influence and net positive influence of these seed node sets selected in the PIM problem. For a certain seed node set, its non-polar influence is the sum of its positive influence and its negative influence, and its net positive influence is its positive influence minus its negative influence. In the experiments, we adopt WC model on the Epinions and Slashdot datasets.

**Figure 8:**
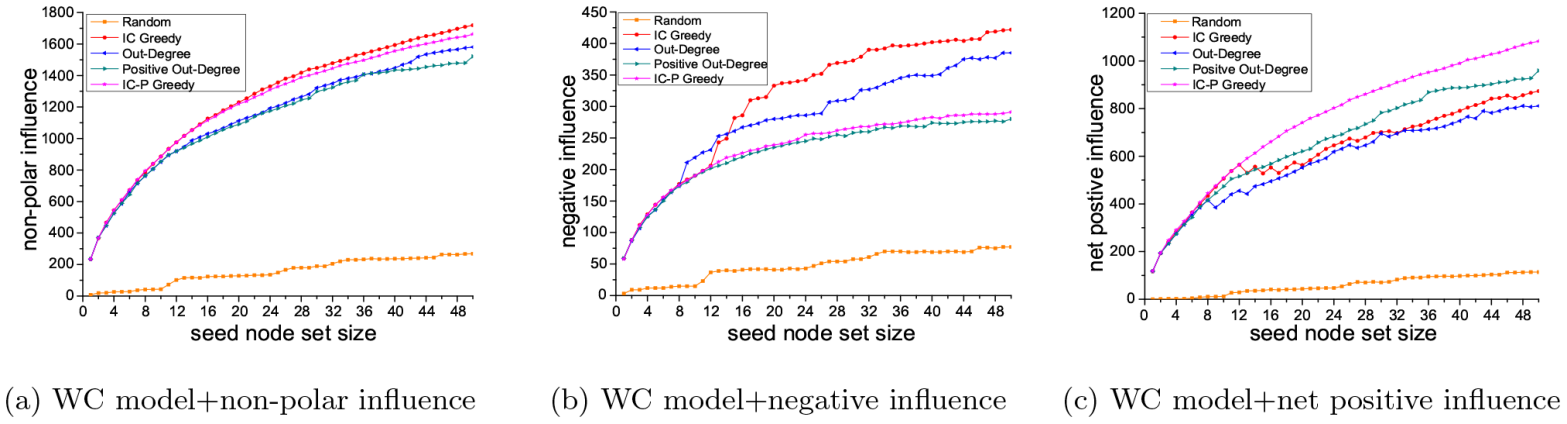
Results on Epinions dataset.

**Figure 9:**
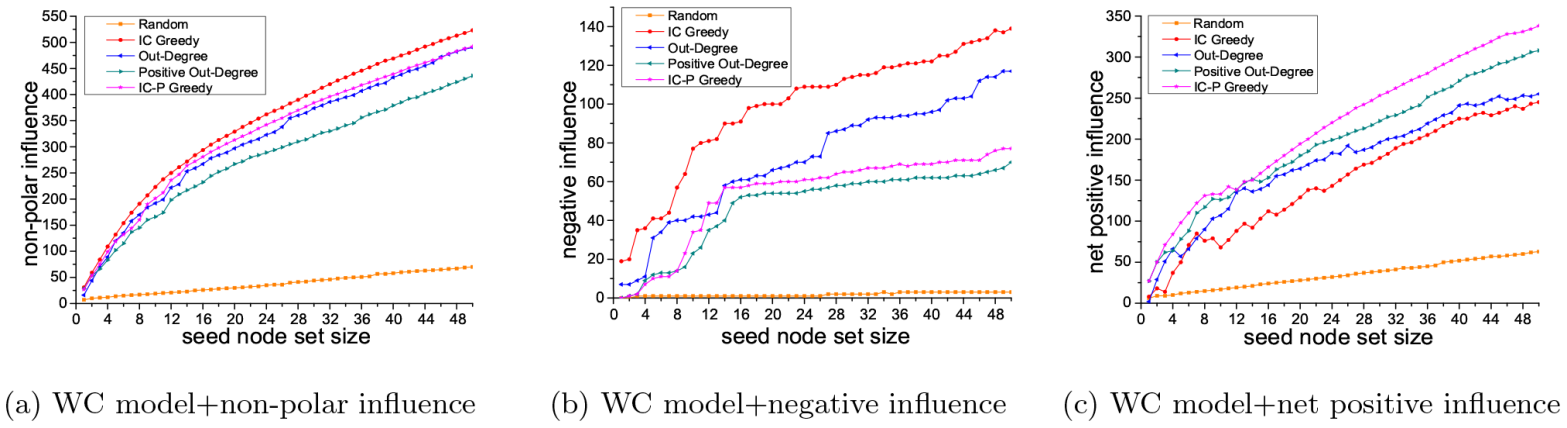
Results on Slashdot dataset.

Figure 8 and Figure 9 show the non-polar influence, negative influence and net positive influence of the seed node sets selected by the five methods on the two datasets. For some applications of the viral marketing, the best solution may be to select the node set with largest positive influence and lowest negative influence. Since we have demonstrated the competent performance of our method on obtaining the largest positive influence in Figure 3 and Figure 4, here, we first focus on the negative influence of our method compared with the others. On Epinions dataset, as shown in Figure 8(b), the negative influence of seed node set selected by our method is 45% lower than that by IC Greedy, 32.3% lower than that by Out-Degree and is close to that by Positive Out-Degree. On Slashdot dataset, see Figure 9(b), the negative influence seed node set selected by our method is 80.5% lower than that by IC Greedy, 51.9% lower than that by Out-Degree and is slightly higher than that by Positive Out-Degree. Another measurement which can give the most straightforward evaluation is the net positive influence. Form Figure 8(c) we can see, on Epinions dataset, compared to IC Greedy and Positive Out-Degree, our method is 23.9% and 12.9% better in terms of the net positive influence. And on Slashdot dataset, Figure 9(c), comparing to IC Greedy and Positive Out-Degree, our method is 38.0% and 9.7% better. From Figure 8(a) and Figure 9(a), we can see that the non-polar of the seed node set selected by IC Greedy is higher than that of our method. But our method can find the seed node set with higher positive influence and net positive influence. In many applications of viral marketing, maximizing non-polar influence may not be the goal. Our results indicate the proposed method is the best solution for viral marketing with different objectives (like maximize positive influence or net positive influence) among all the compared methods.

Note that the solution for the net positive effect is not the optimal solution. Net positive influence maximization (NPIM) problem is a very interesting and important problem. Because the objective function of this problem is not monotone and not submodular under our IC-P model, so we did not use greedy algorithm to solve the problem. In live-edge process for the IC-P model, after coin flipping events and live edge selecting events, each edge will have an outcome (live or blocked). Based on Claim 1, when a new positive node v comes, some shortest live paths whose polarity is positive may reach some nodes, and these nodes will be activated to be positive. Some other shortest live paths whose polarity is negative may reach some other nodes, and those nodes will be activated to be negative. We can not measure whether the number of new positive nodes is larger than new negative nodes, so the objective function of net positive influence maximization problem is not monotone under IC-P model. Similarly, this situation also exists in the proof of submodular. This paper mainly focuses on the PIM problem and NIM problem. For NPIM problem, we only propose it and do preliminary study on it. The specific diffusion model and proof for the NPIM problem are remained as further work.

At last, we discuss the size setting of the seed set. In current studies including our work, the size of seed node set is set between 20 and 50. All these works did not study the impact of seed set size. For deeper studies on influence maximization, it is worth to investigate in more detail about the impact of seed size, so it would be a good direction to look into more. In this paper, because of the efficiency limitation of our proposed greedy algorithm, we do not investigate this problem in our method currently. Here, we study the impact of seed set size using two methods, out-degree and positive out-degree, for PIM problem on Slashdot and Epinions datasets. The size of seed node set is set to be 1000. Then we analyze the positive influence with different sizes of seed node set, ranging from 1 to 1000.

**Figure 10:**
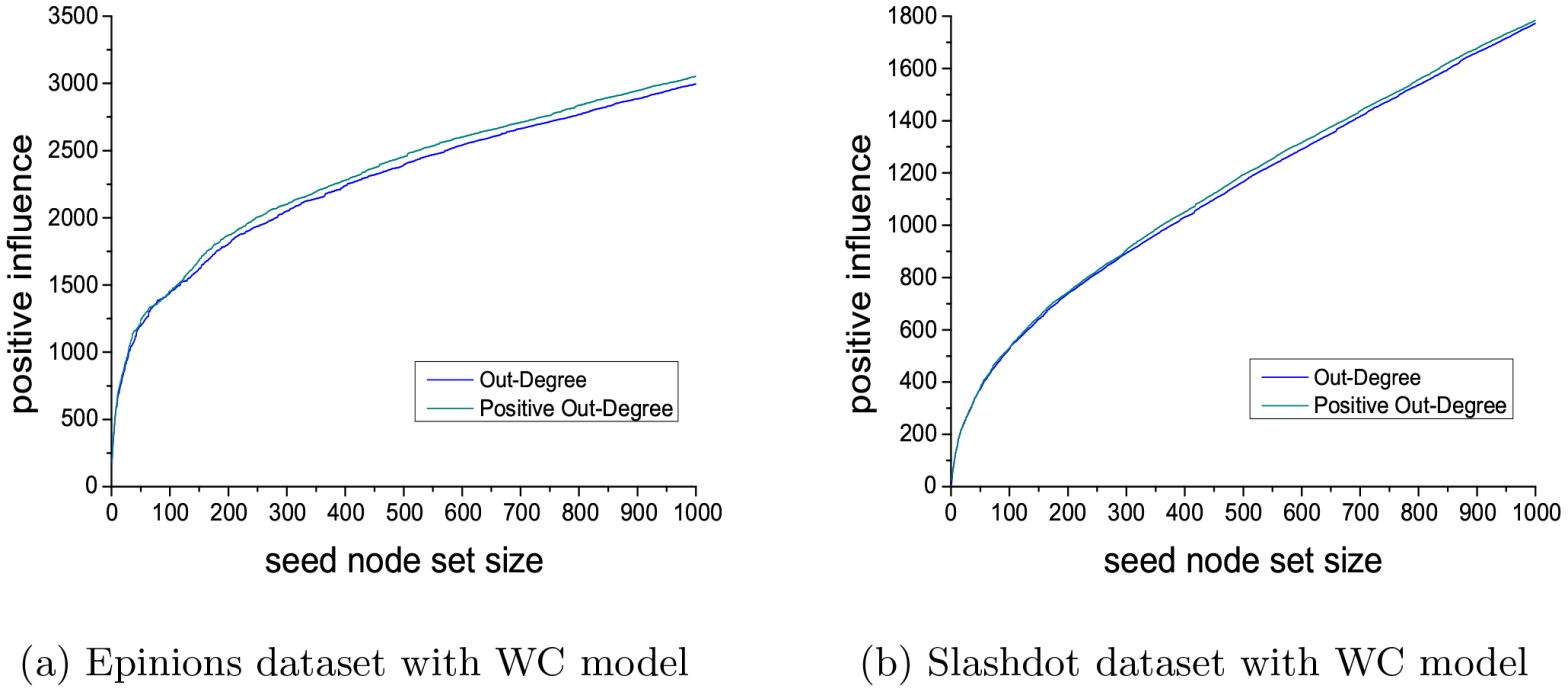
Results for PIM problem (seed node set size is 1000).

Figure 10 shows the performance concerning the PIM problem, using out-degree and positive out-degree with WC model on two datasets. We can see, with the size growth of seed node set, the positive influence increases, but the increase rate slows down. In the application scenarios, the size of seed node set is the cost and positive influence is the gain. The size of seed node set should be set by considering both the limitation of cost and the expectation of gain. Currently, we only do a preliminary study about this problem in this paper, and we will do deeper research along this direction in further work.

## Conclusion

In this paper, we have studied influence maximization in signed social networks, and proposed the polarity-related influence maximization (PRIM) problem which aims to find the node set with maximum positive influence or maximum negative influence in signed social networks. We divided the PRIM problem into two sub-problems, positive influence maximization (PIM) problem and negative influence maximization (NIM) problem. To address these problems, we first extended the standard independent cascade model to the signed social networks, and proposed a new polarity-related Independent Cascade diffusion model (IC-P model). Then, we proved that the influence function of the PIM and NIM problem under the IC-P diffusion model is monotone and submodular, This implies that a greedy approximation algorithm can solve the PIM and NIM problem within a ratio of 1-1/e. Finally, we demonstrate the superiority of our algorithm compared with the IC greedy based on standard IC model and other heuristic algorithms through simulations on two online signed social networks.

Several challenges and future directions remain. One challenge is to improve our greedy algorithm to further reduce its running time. For today's large scale social networks, even this solution is computationally expensive. Therefore, reducing running time is necessary. The methods used for improving the original greedy algorithm for unsigned social networks in the literature are of great use for reference. Another future direction is to study influence maximization in signed social networks under other diffusion models, such as the Epidemic model and the Linear Threshold model.
